# Optimizing cognitive health and emotional well-being through daytime napping: current insights and future directions

**DOI:** 10.1097/MS9.0000000000003968

**Published:** 2025-10-13

**Authors:** Shayan Shadab, Tameen Khursheed, Ahmed Asad Raza, Abedin Samadi

**Affiliations:** Department of Medicine, Jinnah Sindh Medical University, Karachi, Pakistan

**Keywords:** circadian rhythm, cognitive function, mood regulation, napping, sleep deprivation

## Abstract

Daytime napping offers numerous benefits, including cognitive enhancement, mood regulation, and improved well-being. Averaging around an hour, naps help recover from sleep deprivation and prepare for future sleep deficits. They significantly boost alertness, sustained attention, declarative memory, and learning capacity. Daytime naps boost alertness, memory, and resilience while improving mood. A 60-min nap aids learning and counters sleep deficits, enhancing cognitive recovery. Napping also aids in cellular regeneration and reduces cognitive decline, particularly in children, adolescents, and older adults. The internal biological clock supports napping, aligning with natural circadian rhythms. Additionally, naps mitigate stress, improve emotional resilience, and enhance performance and positive psychology. This study highlights the need for further research on the long-term impacts of regular napping, optimal nap durations, and cultural influences on napping practices.

## Introduction

Daytime nap is relatively a brief respite shorter than a full sleep cycle usually taken to recover from sleep deprivation, prepare for completing future sleep deficits and more importantly relaxation. Moreover it has been shown to improve alertness, reduce fatigue, and positively impact cognitive functions. The duration of a nap on average is around an hour varying from a few minutes to several hours^[1]^. According to a study, naps taken mid-afternoon can enhance alertness, sustained attention, declarative memory, and the capacity to attain new knowledge^[2]^. Sleep also contributes to cognitive resilience by reducing oxidative stress and preventing synaptic overload. Furthermore, it plays a vital role in restoring bodily equilibrium, supporting immune function, neural repair, and metabolic regulation. When sleep is disrupted, these restorative mechanisms are impaired, increasing vulnerability to illness^[3]^.

Other than the cognitive functions, physiological processes and sleep requirements also are affected that varies with age^[4]^.

The individuals who are sleep deprived are mostly children and adolescents significantly affecting their health and is a major public health issue affecting their development and functioning, including mood, behavior, physical health, quality of life, academic performance and most importantly impair cognition and increase the risk of behavioral and emotional disturbances, and criminality in later life^[5]^. In order to evade these issues, napping has been considered a possible therapeutic intervention that can also cure inadequate nocturnal sleep^[6]^. Furthermore older adults are more likely to nap due to changes in social and physical activities and age-related health changes^[7]^.

**HIGHLIGHTS**

- Napping boosts alertness, memory, and emotional resilience.
- Short naps (<30 mins) enhance cognition without harming night sleep.
- Moderate naps (30–90 mins) support brain health and mood.
- Circadian rhythms favor naps between 1–5 PM for peak benefit.
- Overlong naps (>60 mins) may impair sleep and speed cognitive decline.

This review aims to synthesize current evidence on the cognitive and emotional benefits of daytime napping, explore the underlying biological mechanisms, and evaluate the influence of nap duration and frequency across the lifespan. It also seeks to identify potential risks and highlight gaps in knowledge that warrant further research.

### Methodology

This narrative review was conducted to explore the current literature on the cognitive, emotional, and physiological impacts of daytime napping across various age groups. A thorough search was performed using electronic databases such as PubMed, Scopus, Web of Science, and Google Scholar. The search focused on articles published between 2000 and 2024 using relevant keywords, including “daytime nap,” “cognitive function,” “emotional wellbeing,” “circadian rhythm,” “sleep deprivation,” and “nap duration.”

To ensure relevance and quality, studies were selected based on specific criteria. Included in the review were peer-reviewed articles published in English that investigated the effects of daytime napping on human participants. Eligible studies examined aspects such as cognitive performance, mood regulation, physiological processes, or neurological mechanisms associated with napping. Both experimental and observational studies, as well as systematic reviews, were considered if they provided insights into the relationship between nap characteristics – such as duration and frequency – and health outcomes.

Studies were excluded if they were non-peer-reviewed (e.g., editorials, opinion pieces), focused solely on nocturnal sleep without discussing daytime napping, or were conducted exclusively on animals unless they offered significant translational relevance to human physiology. Additionally, studies lacking methodological clarity or specific outcomes related to napping were also excluded from the final selection.

The included studies were analyzed thematically to synthesize current insights, identify knowledge gaps, and guide future research directions on the role of napping in cognitive and emotional wellbeing.

AI tools (e.g., ChatGPT) were used in accordance with TITAN guidelines to assist with language refinement and structural clarity; all content was reviewed and verified by the authors for accuracy and integrity^[8]^.

## Sleep and cognitive health

Sleep is crucial for the body’s and brain’s recovery processes, aiding in cellular regeneration, cognitive function, and restoring energy and attention. Regular napping may offer resilience to cognitive impairment, especially for those who struggle with nighttime sleep, by compensating for sleep loss^[9]^. The “glymphatic” (glial-lymphatic) system has been identified as essential for removing waste from the brain’s interstitial spaces, with research showing that its activity increases during sleep^[10]^. The internal biological clock, located in the hypothalamus’s suprachiasmatic nuclei, regulates wakefulness, sleep intervals, body temperature, blood pressure, hormonal levels, alertness, mood, and cognitive abilities, which fluctuate throughout the day. Human cognitive function follows a circadian rhythm, peaking in the early evening and declining in the afternoon^[11]^. The biological clock encourages two sleep cycles: one from 1 to 5 a.m. and another from 1 to 5 p.m., indicating an innate propensity for naps in the early afternoon^[12]^. Learning is improved in the afternoon when sleep is divided into a nocturnal sleep and a daytime nap^[6]^. A midday nap can improve brain communication related to motor skills, while a full night’s sleep enhances planning and problem-solving communication^[13]^.

## Stress levels and emotional resilience

Napping reduces daytime drowsiness and helps recover from prolonged sleep deprivation, extending total sleep duration and enhancing visuospatial processing and motor procedural learning. Eliminating chronic sleep deprivation, or “sleep debt,” can improve performance due to additional sleep^[14]^. Longer naps (31–60 and >60 min) and frequent naps (5–7 times/week) are associated with better academic performance and positive psychology^[5]^. Moderate napping (30–90 min) is linked to reduced cognitive decline risk and better cognitive performance^[9]^. Longer sleep may improve mental states in comparison to no naps^[4]^. Small midday naps slow biological age and cognitive decline^[9]^. Furthermore, greater neuromuscular performance during midday naps may be linked to increased cortisol levels, which can reduce stress^[15]^.

## Mood regulation and subjective well-being

Napping lightens up the mood by augmenting the positive mood and alleviating negative moods in all individuals, whether they are depressive or not. On the contrary the sleep-deprived or disturbed individuals have mood disorders and have less emotional processing, making them have less intense emotions compared to healthy sleepers^[16]^. During the napping phase, specific neurophysiological processes occur, which have a positive influence on patients’ emotions, including their subjective well-being^[17]^. Napping also boosts up energy availability and heightens antioxidants’ response post exercise, strengthening resistance to oxidative stress; all happening due to increased glucose levels^[18]^. Overall, napping is beneficial for the individuals making them happier and having better grit, self-control, reduced internalizing behavior problems, higher verbal IQs, and better academic performances^[5]^.

## Biological mechanisms

Cognitive function is assessed through domains such as memory, attention, language, visuospatial function, and executive function^[19]^. Rapid eye movement (REM) sleep is crucial for cognitive restoration, memory consolidation, and motor skill learning, while non-rapid eye movement (NREM) sleep aids physical repair. Daytime napping facilitates muscle relaxation and structural and functional recuperation, with the most significant benefits observed in naps lasting 30 to 60 min. However, it may take roughly 60 min after waking to fully overcome sleep inertia and achieve peak physical performance^[17]^. Slow-wave sleep (SWS), or deeper sleep, is essential for cerebral recovery. A 90-min nap may complete a sleep cycle (NREM + REM), reducing sleep inertia severity since REM sleep is lighter^[20]^. The propensity for daytime napping is biologically driven, with a peak during the circadian dip in alertness typically occurring between 2:00 and 4:00 PM. Sleep inertia, marked by grogginess and confusion post-nap, depends on the amount of SWS; shorter naps (20–30 min) minimize this inertia^[12]^. In addition to the short-term benefits discussed, increasing evidence supports the role of daytime napping in promoting long-term neurocognitive health. Specifically, naps may contribute to the sustained improvement of memory consolidation, which is essential for learning and adaptive behavior^[2]^. Executive functions – such as working memory, cognitive flexibility, planning, and inhibitory control – are also positively influenced by the restorative effects of napping, possibly through enhanced prefrontal cortex regulation and reduction of cognitive fatigue^[19]^. Improvements in visual-spatial ability have been observed in both younger and older adults following structured napping, pointing to its role in preserving spatial reasoning and sensorimotor integration^[21]^. Moreover, while mood regulation is well documented, there is a growing interest in the impact of napping on anxiety levels, with preliminary findings suggesting that naps may reduce anxiety symptoms by modulating cortisol levels and enhancing parasympathetic tone^[15,16]^. Most importantly, the neurophysiological mechanisms triggered during naps – such as increased spindle activity, hippocampal reactivation, synaptic pruning, and dynamic shifts in excitation/inhibition balance – are all markers of heightened neuroplasticity^[2,21]^. This biological adaptability is essential not only for memory and learning but also for emotional regulation and brain health across the lifespan^[17]^.

**Figure 1.**
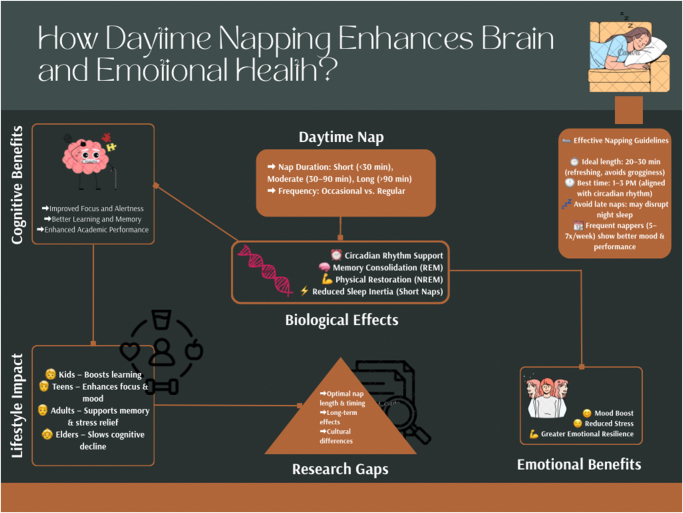
Visual overview of how daytime napping supports cognitive and emotional health through biological effects, optimal timing, and age-related impacts.

An integrated summary of these findings is illustrated in Fig. 1, which highlights the multidimensional benefits of daytime napping and identifies critical gaps in current knowledge.

## Napping across the lifespan

Napping prevalence varies with age^[20]^. Sleep insufficiency is a significant public health concern, especially in children and adolescents, impacting development, cognition, school performance, mood, behavior, physical health, and quality of life. Excessive daytime sleepiness in children is linked to lower cognitive abilities and increased emotional and behavioral issues, even leading to adult criminality^[5]^. Older adults benefit from short or moderate-duration naps (<90 min), improving cognitive health^[7]^. A 2023 longitudinal study in *Sleep Health*, Paz *et al* (*n* > 10 000 adults followed over 5 years) found that habitual short daytime naps (< 30 min) were associated with stable cognitive performance in mid-to-late life, whereas prolonged napping (>60 min) correlated with accelerated cognitive decline, underscoring the importance of moderation in napping habits for long-term brain health^[22]^.

## Daytime napping and nighttime sleep

While daytime napping improves mood, alertness, and performance, naps can be detrimental to subsequent nocturnal sleep (i.e., increased sleep onset latency and sleep fragmentation with a decreased sleep efficiency), probably due to a decreased homeostatic sleep pressure. While frequent late-evening naps correlate with nighttime sleep fragmentation and reduced quality, early daytime naps in moderation show no adverse effects on nocturnal sleep integrity^[22]^.

## Physiological and structural effects of daily naps

Plasticity increases, shown by the enhanced Excitation/Inhibition (E/I) balance in early visual areas during NREM sleep, in a learning-independent manner. Stabilization during REM sleep, marked by a decrease in E/I balance, occurs in a learning-dependent manner. NREM contributes to performance gains, while REM stabilizes learning. Synapses involved in visual learning are reactivated during NREM, with important ones maintained and less important ones pruned during REM. Sigma (13–16 Hz) and delta (1–4 Hz) activity during NREM, and theta (5–7 Hz) during REM, facilitate learning during sleep^[21]^. Hippocampal activation increased following the nap, suggestive of restored hippocampal capacity, and was positively correlated with spindle count. Functional connectivity between the hippocampus and neocortex has been associated with fast spindles, which might promote the transfer of reactivated information. Both post-nap hippocampal activation and learning change were correlated with the amount of fast spindles during the nap^[2]^.

## Nap duration and BMI

Long daytime napping (> 1 h) demonstrates a bidirectional association with obesity. Physiologically, it elevates nighttime cortisol levels, contributing to abnormal lipid metabolism and fat distribution, while increased sympathetic activity promotes appetite stimulation and fat deposition. As a behavioral factor, it reduces physical activity and calorie expenditure, further increasing obesity risk^[23]^. One meta-analytic evidence further associates prolonged napping with exacerbated nighttime insomnia, sleep fragmentation, and poor sleep quality as an established risk factor for weight gain^[24]^. Obesity adversely impacts neurocognitive pathways through structural brain alterations, leptin/insulin resistance, oxidative stress, cerebrovascular and blood-brain barrier disruption, and chronic neuroinflammation^[25]^. Critically, midlife obesity elevates Alzheimer’s disease risk, while obesity in older adults impairs learning and working memory. Weight management interventions are critical for neuroprotection in aging populations^[26]^.

It is important to note that body mass index (BMI) may also moderate the cognitive benefits associated with spindle activity during naps. While moderate naps enhance spindle generation and memory consolidation^[2]^, obesity-related mechanisms such as insulin resistance, neuroinflammation, and cerebrovascular changes may attenuate spindle efficacy and reduce neuroplasticity^[24,25]^. Moreover, prolonged napping has been associated with both obesity and poor sleep quality^[23]^. These pathways suggest that the cognitive benefits of napping may be blunted in individuals with elevated BMI, warranting further investigation.

## Conclusion

Strategic daytime napping (30–60 min, aligned with circadian rhythms), boosts alertness, strengthens memory, enhances cognitive performance, emotional resilience, physiological restoration, and learning capacity while reducing oxidative stress and supporting neural repair. They further elevate mood, self-regulation, and stress recovery across all life stages. These benefits are critical for children’s academic outcomes, adolescents’ behavioral health, and older adults’ cognitive preservation. However, excessive napping correlates bidirectionally with obesity via elevated nighttime cortisol, appetite dysregulation, reduced physical activity, and sleep fragmentation, while also risking nighttime sleep disruption. While naps offer compensatory benefits for sleep-deprived populations such as children, adolescents, and older adults, their efficacy hinges on moderation and alignment with individual circadian rhythms. Additionally, sex-specific responses to nap duration and timing warrant investigation, as hormonal and circadian differences may influence cognitive and emotional outcomes. Future research should prioritize clarifying napping in men and women, long-term outcomes, and culturally tailored practices to translate these insights into actionable public health strategies.

## Data Availability

No data is available.

## Peer and provenance statement

Externally peer reviewed: not commissioned.
